# *catena*-Poly[[diaqua­cobalt(II)]-μ-4,4′-[1,4-phenyl­enebis(­oxy)]dibutano­ato-κ^4^ *O*,*O*′:*O*′′,*O*′′′]

**DOI:** 10.1107/S1600536810053742

**Published:** 2011-01-08

**Authors:** Ying-Ying Zhao

**Affiliations:** a College of Chemistry and Chemical Engineering, Bohai University, 121013 Jinzhou, Liaoning, People’s Republic of China

## Abstract

In the title coordination polymer, [Co(C_14_H_16_O_6_)(H_2_O)_2_]_*n*_, the Co^II^ ion, situated on a twofold rotation axis, is coordinated by four O atoms from two 4,4′-[1,4-phenyl­enebis(­oxy)]dibutano­ate (*L*) ligands and two water mol­ecules in a highly distorted octa­hedral geometry. Each *L* ligand is situated on an inversion center and bridges two Co^II^ atoms, forming a zigzag polymeric chain propagating in [10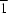]. Inter­molecular O—H⋯O hydrogen bonds further consolidate the crystal packing.

## Related literature

For related structures, see: Dai *et al.* (2009 ▶); Zhu *et al.* (2008 ▶); Li *et al.* (2010 ▶). For the synthesis of 4,4′-[1,4-phenyl­enebis(­oxy)]dibutanoic acid, see: Zhang *et al.* (2009 ▶).
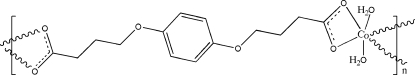


## Experimental

### 

#### Crystal data

[Co(C_14_H_16_O_6_)(H_2_O)_2_]
*M* *_r_* = 375.23
Monoclinic, 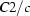

*a* = 28.835 (4) Å
*b* = 5.4057 (8) Å
*c* = 10.6425 (16) Å
β = 98.126 (2)°
*V* = 1642.2 (4) Å^3^
*Z* = 4
Mo *K*α radiation
μ = 1.08 mm^−1^
*T* = 296 K
0.18 × 0.16 × 0.10 mm

#### Data collection

Bruker APEXII CCD area-detector diffractometer
Absorption correction: multi-scan (*SADABS*; Sheldrick, 1996 ▶) *T*_min_ = 0.829, *T*_max_ = 0.900
5204 measured reflections
1459 independent reflections
1343 reflections with *I* > 2σ(*I*)
*R*_int_ = 0.032

#### Refinement

*R*[*F* ^2^ > 2σ(*F* ^2^)] = 0.028
*wR*(*F* ^2^) = 0.080
*S* = 1.00
1459 reflections
105 parameters
H-atom parameters constrained
Δρ_max_ = 0.26 e Å^−3^
Δρ_min_ = −0.25 e Å^−3^

### 

Data collection: *APEX2* (Bruker, 2005 ▶); cell refinement: *SAINT* (Bruker, 2005 ▶); data reduction: *SAINT*; program(s) used to solve structure: *SHELXS97* (Sheldrick, 2008 ▶); program(s) used to refine structure: *SHELXL97* (Sheldrick, 2008 ▶); molecular graphics: *SHELXTL* (Sheldrick, 2008 ▶); software used to prepare material for publication: *SHELXTL*.

## Supplementary Material

Crystal structure: contains datablocks global, I. DOI: 10.1107/S1600536810053742/cv5012sup1.cif

Structure factors: contains datablocks I. DOI: 10.1107/S1600536810053742/cv5012Isup2.hkl

Additional supplementary materials:  crystallographic information; 3D view; checkCIF report

## Figures and Tables

**Table 1:** Hydrogen-bond geometry (Å, °)

*D*—H⋯*A*	*D*—H	H⋯*A*	*D*⋯*A*	*D*—H⋯*A*
O4—H9⋯O2^i^	0.85	1.88	2.7335 (19)	176
O4—H8⋯O1^ii^	0.85	1.89	2.740 (2)	175

Symmetry codes: (i) 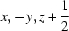; (ii) 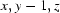.

**Fig. 1.**
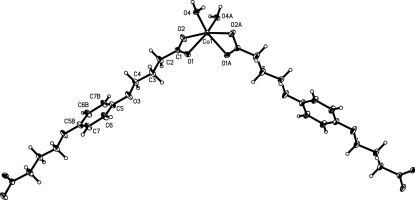
A portion of the crystal structure of (I) showing the atomic labeling and 30% probability displacement ellopsoids [symmetry codes: (A) -x, y, -z + 3/2; (B) -x + 1/2, -y + 5/2, -z + 1].

**Fig. 2.**
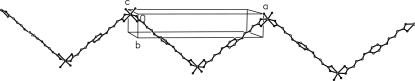
A portion ot the crystal packing of (I) showing the polymeric one-dimensional zigzag chain.

## supplementary crystallographic information

### Comment

Benzene-1,4-dioxydiacetic acid is an important biologically active compound commonly used in herbicides and plant-growth agents. Two phenoxyacetate groups have versatile bonding modes to metal ions and easily forms complexes (Dai *et al.*, 2009; Zhu *et al.*, 2008; Li *et al.*, 2010). Benzene-1,4-dioxydibutanoic acid is an interesting dicarboxylate ligand. To our knowledge, there have been no reports about its coordination compounds. Recently, we obtained the title cobalt polymer (I), and its crystal structure is reported here.

In the structure of (I), each cobalt(II) atom is coordinated by four oxygen atoms from two benzene-1,4-dioxydibutyrate ligands and two water molecules, displaing highly distorted octahedral geometry (Fig. 1). Each ligand situated on an inversion center bridges two cobalt(II) centers to form polymeric zigzag chain propagated in direction [10–1] (Fig. 2). Intermolecular O—H···O hydrogen bonds (Table 1) consolidate further the crystal packing.

### Experimental

The ligand was prepared according to the literature method (Zhang *et al.*, 2009). A mixture of CoSO4 (0.5 mmol), benzene-1,4-dioxydiacetic acid (0.5 mmol), NaOH (1 mmol) and H_2_O (12 ml) was placed in a 23 ml Teflon reactor, which was heated at 423 K for three days and then cooled to room temperature. Single crystals were obtained after washing with water and drying in air.

### Refinement

All H atoms were placed in idealized positions (O—H = 0.85 Å and C—H = 0.93–0.97 Å) and refined as riding atoms with *U*_iso_(H) = 1.2*U*_eq_(C) and *U*_iso_(H) = 1.5*U*_eq_(O).

### Crystal data

**Table:**

[Co(C_14_H_16_O_6_)(H_2_O)_2_]	*F*(000) = 780
*M**_r_* = 375.23	*D*_x_ = 1.518 Mg m^−^^3^
Monoclinic, *C*2/*c*	Mo *K*α radiation, λ = 0.71073 Å
Hall symbol: -C 2yc	Cell parameters from 2107 reflections
*a* = 28.835 (4) Å	θ = 2.9–27.0°
*b* = 5.4057 (8) Å	µ = 1.08 mm^−^^1^
*c* = 10.6425 (16) Å	*T* = 296 K
β = 98.126 (2)°	Acicular, red
*V* = 1642.2 (4) Å^3^	0.18 × 0.16 × 0.10 mm
*Z* = 4	

### Data collection

**Table:**

Bruker APEXII CCD area-detector diffractometer	1459 independent reflections
Radiation source: fine-focus sealed tube	1343 reflections with *I* > 2σ(*I*)
graphite	*R*_int_ = 0.032
φ and ω scans	θ_max_ = 25.0°, θ_min_ = 2.9°
Absorption correction: multi-scan (*SADABS*; Sheldrick, 1996)	*h* = −34→34
*T*_min_ = 0.829, *T*_max_ = 0.900	*k* = −4→6
5204 measured reflections	*l* = −12→12

### Refinement

**Table:**

Refinement on *F*^2^	Primary atom site location: structure-invariant direct methods
Least-squares matrix: full	Secondary atom site location: difference Fourier map
*R*[*F*^2^ > 2σ(*F*^2^)] = 0.028	Hydrogen site location: inferred from neighbouring sites
*wR*(*F*^2^) = 0.080	H-atom parameters constrained
*S* = 1.00	*w* = 1/[σ^2^(*F*_o_^2^) + (0.0516*P*)^2^ + 0.6799*P*] where *P* = (*F*_o_^2^ + 2*F*_c_^2^)/3
1459 reflections	(Δ/σ)_max_ < 0.001
105 parameters	Δρ_max_ = 0.26 e Å^−^^3^
0 restraints	Δρ_min_ = −0.25 e Å^−^^3^

### Special details

**Table:**

Geometry. All e.s.d.'s (except the e.s.d. in the dihedral angle between two l.s. planes) are estimated using the full covariance matrix. The cell e.s.d.'s are taken into account individually in the estimation of e.s.d.'s in distances, angles and torsion angles; correlations between e.s.d.'s in cell parameters are only used when they are defined by crystal symmetry. An approximate (isotropic) treatment of cell e.s.d.'s is used for estimating e.s.d.'s involving l.s. planes.
Refinement. Refinement of *F*^2^ against ALL reflections. The weighted *R*-factor *wR* and goodness of fit *S* are based on *F*^2^, conventional *R*-factors *R* are based on *F*, with *F* set to zero for negative *F*^2^. The threshold expression of *F*^2^ > σ(*F*^2^) is used only for calculating *R*-factors(gt) *etc*. and is not relevant to the choice of reflections for refinement. *R*-factors based on *F*^2^ are statistically about twice as large as those based on *F*, and *R*- factors based on ALL data will be even larger.

### Fractional atomic coordinates and isotropic or equivalent isotropic displacement parameters (Å^2^)

**Table:**

	*x*	*y*	*z*	*U*_iso_*/*U*_eq_	
Co1	0.0000	0.16787 (6)	0.7500	0.02397 (16)	
O1	0.05242 (5)	0.4611 (3)	0.74548 (12)	0.0332 (3)	
O2	0.03451 (5)	0.2091 (3)	0.58828 (13)	0.0399 (4)	
O3	0.18305 (5)	1.0014 (3)	0.61283 (14)	0.0458 (4)	
O4	0.04626 (5)	−0.0762 (3)	0.84698 (12)	0.0322 (3)	
H8	0.0494	−0.2161	0.8127	0.048*	
H9	0.0419	−0.1122	0.9221	0.048*	
C1	0.05869 (7)	0.3883 (4)	0.63762 (18)	0.0280 (4)	
C2	0.09363 (8)	0.5074 (5)	0.5643 (2)	0.0425 (6)	
H2A	0.1135	0.3785	0.5375	0.051*	
H2B	0.0766	0.5807	0.4882	0.051*	
C3	0.12459 (8)	0.7026 (4)	0.6324 (2)	0.0374 (5)	
H3A	0.1437	0.6303	0.7056	0.045*	
H3B	0.1054	0.8315	0.6622	0.045*	
C4	0.15584 (7)	0.8131 (4)	0.5448 (2)	0.0357 (5)	
H4A	0.1761	0.6868	0.5174	0.043*	
H4B	0.1371	0.8823	0.4703	0.043*	
C5	0.21584 (7)	1.1201 (4)	0.55206 (19)	0.0338 (5)	
C6	0.24618 (8)	1.2756 (5)	0.6269 (2)	0.0413 (6)	
H6	0.2437	1.2927	0.7126	0.050*	
C7	0.28037 (8)	1.4066 (4)	0.5754 (2)	0.0391 (5)	
H7	0.3006	1.5115	0.6262	0.047*	

### Atomic displacement parameters (Å^2^)

**Table:**

	*U*^11^	*U*^22^	*U*^33^	*U*^12^	*U*^13^	*U*^23^
Co1	0.0283 (2)	0.0205 (2)	0.0252 (2)	0.000	0.01100 (15)	0.000
O1	0.0445 (8)	0.0282 (8)	0.0301 (8)	−0.0084 (6)	0.0157 (6)	−0.0030 (6)
O2	0.0489 (9)	0.0464 (10)	0.0268 (8)	−0.0265 (8)	0.0139 (7)	−0.0082 (7)
O3	0.0502 (9)	0.0547 (11)	0.0350 (8)	−0.0326 (8)	0.0147 (7)	−0.0047 (7)
O4	0.0425 (8)	0.0297 (8)	0.0260 (7)	0.0093 (6)	0.0100 (6)	0.0018 (6)
C1	0.0286 (10)	0.0300 (11)	0.0259 (10)	−0.0043 (8)	0.0060 (8)	0.0024 (8)
C2	0.0476 (12)	0.0487 (15)	0.0343 (12)	−0.0212 (11)	0.0163 (10)	−0.0041 (10)
C3	0.0386 (12)	0.0432 (14)	0.0316 (11)	−0.0174 (10)	0.0097 (9)	−0.0016 (10)
C4	0.0356 (12)	0.0390 (14)	0.0333 (12)	−0.0125 (10)	0.0076 (9)	0.0007 (9)
C5	0.0338 (11)	0.0372 (13)	0.0308 (11)	−0.0119 (9)	0.0062 (9)	0.0047 (9)
C6	0.0492 (13)	0.0499 (14)	0.0258 (11)	−0.0216 (11)	0.0084 (10)	−0.0015 (10)
C7	0.0410 (12)	0.0446 (13)	0.0314 (11)	−0.0206 (11)	0.0039 (9)	−0.0022 (10)

### Geometric parameters (Å, °)

**Table:**

Co1—O4^i^	2.0485 (13)	C2—H2A	0.9700
Co1—O4	2.0485 (13)	C2—H2B	0.9700
Co1—O2	2.1181 (14)	C3—C4	1.508 (3)
Co1—O2^i^	2.1181 (14)	C3—H3A	0.9700
Co1—O1^i^	2.1954 (13)	C3—H3B	0.9700
Co1—O1	2.1955 (14)	C4—H4A	0.9700
O1—C1	1.251 (2)	C4—H4B	0.9700
O2—C1	1.263 (2)	C5—C6	1.382 (3)
O3—C5	1.377 (2)	C5—C7^ii^	1.384 (3)
O3—C4	1.420 (2)	C6—C7	1.388 (3)
O4—H8	0.8499	C6—H6	0.9300
O4—H9	0.8499	C7—C5^ii^	1.384 (3)
C1—C2	1.503 (3)	C7—H7	0.9300
C2—C3	1.501 (3)		
			
O4^i^—Co1—O4	99.80 (8)	C1—C2—H2B	108.2
O4^i^—Co1—O2	90.32 (5)	H2A—C2—H2B	107.3
O4—Co1—O2	97.47 (6)	C2—C3—C4	110.28 (18)
O4—Co1—O2^i^	90.33 (5)	C2—C3—H3A	109.6
O2—Co1—O2^i^	167.91 (9)	C4—C3—H3A	109.6
O4—Co1—O1^i^	148.79 (5)	C2—C3—H3B	109.6
O2^i^—Co1—O1^i^	60.14 (5)	C4—C3—H3B	109.6
O4^i^—Co1—O1	148.79 (5)	H3A—C3—H3B	108.1
O4—Co1—O1	94.29 (6)	O3—C4—C3	107.70 (17)
O2—Co1—O1	60.15 (5)	O3—C4—H4A	110.2
O2^i^—Co1—O1	110.23 (6)	C3—C4—H4A	110.2
O1^i^—Co1—O1	87.57 (8)	O3—C4—H4B	110.2
C5—O3—C4	117.46 (16)	C3—C4—H4B	110.2
Co1—O4—H8	117.3	H4A—C4—H4B	108.5
Co1—O4—H9	116.6	O3—C5—C6	115.70 (19)
H8—O4—H9	103.9	O3—C5—C7^ii^	124.50 (19)
O1—C1—O2	118.67 (17)	C6—C5—C7^ii^	119.8 (2)
O1—C1—C2	122.41 (18)	C5—C6—C7	120.7 (2)
O2—C1—C2	118.92 (17)	C5—C6—H6	119.7
C3—C2—C1	116.56 (18)	C7—C6—H6	119.7
C3—C2—H2A	108.2	C5^ii^—C7—C6	119.5 (2)
C1—C2—H2A	108.2	C5^ii^—C7—H7	120.2
C3—C2—H2B	108.2	C6—C7—H7	120.2
			
O4^i^—Co1—O1—C1	−21.60 (18)	Co1—O1—C1—C2	−179.14 (19)
O4—Co1—O1—C1	95.34 (12)	Co1—O2—C1—O1	−1.4 (2)
O2—Co1—O1—C1	−0.82 (12)	Co1—O2—C1—C2	179.07 (17)
O2^i^—Co1—O1—C1	−172.73 (12)	O1—C1—C2—C3	6.2 (3)
O1^i^—Co1—O1—C1	−115.86 (13)	O2—C1—C2—C3	−174.3 (2)
C1^i^—Co1—O1—C1	−143.01 (11)	C1—C2—C3—C4	−177.1 (2)
O4^i^—Co1—O2—C1	170.22 (13)	C5—O3—C4—C3	177.08 (19)
O4—Co1—O2—C1	−89.86 (13)	C2—C3—C4—O3	178.05 (18)
O2^i^—Co1—O2—C1	39.90 (12)	C4—O3—C5—C6	−170.9 (2)
O1^i^—Co1—O2—C1	75.53 (13)	C4—O3—C5—C7^ii^	9.4 (3)
O1—Co1—O2—C1	0.81 (12)	O3—C5—C6—C7	−179.4 (2)
C1^i^—Co1—O2—C1	69.9 (2)	C7^ii^—C5—C6—C7	0.3 (4)
Co1—O1—C1—O2	1.4 (2)	C5—C6—C7—C5^ii^	−0.3 (4)

Symmetry codes: (i) −*x*, *y*, −*z*+3/2; (ii) −*x*+1/2, −*y*+5/2, −*z*+1.

### Hydrogen-bond geometry (Å, °)

**Table:**

*D*—H···*A*	*D*—H	H···*A*	*D*···*A*	*D*—H···*A*
O4—H9···O2^iii^	0.85	1.88	2.7335 (19)	176
O4—H8···O1^iv^	0.85	1.89	2.740 (2)	175

Symmetry codes: (iii) *x*, −*y*, *z*+1/2; (iv) *x*, *y*−1, *z*.
